# Effects of Sleep Quality on the Association between Problematic Mobile Phone Use and Mental Health Symptoms in Chinese College Students

**DOI:** 10.3390/ijerph14020185

**Published:** 2017-02-14

**Authors:** Shuman Tao, Xiaoyan Wu, Yukun Zhang, Shichen Zhang, Shilu Tong, Fangbiao Tao

**Affiliations:** 1 Department of Maternal, Child & Adolescent Health, School of Public Health, Anhui Medical University, Hefei 230032, China; shumantao@126.com (S.T.); xywu85@126.com (X.W.); zhangyukun1106@gmail.com (Y.Z.); season0724@gmail.com (S.Z.); 2 Anhui Provincial Key Laboratory of Population Health & Aristogenics, Hefei 230032, China; s.tong@qut.edu.au; 3 School of Public Health and Social Work, Queensland University of Technology, Kelvin Grove, QLD 4059, Australia

**Keywords:** cellular phone, cross-sectional studies, sleep, anxiety, depression

## Abstract

Problematic mobile phone use (PMPU) is a risk factor for both adolescents’ sleep quality and mental health. It is important to examine the potential negative health effects of PMPU exposure. This study aims to evaluate PMPU and its association with mental health in Chinese college students. Furthermore, we investigated how sleep quality influences this association. In 2013, we collected data regarding participants’ PMPU, sleep quality, and mental health (psychopathological symptoms, anxiety, and depressive symptoms) by standardized questionnaires in 4747 college students. Multivariate logistic regression analysis was applied to assess independent effects and interactions of PMPU and sleep quality with mental health. PMPU and poor sleep quality were observed in 28.2% and 9.8% of participants, respectively. Adjusted logistic regression models suggested independent associations of PMPU and sleep quality with mental health (*p* < 0.001). Further regression analyses suggested a significant interaction between these measures (*p* < 0.001). The study highlights that poor sleep quality may play a more significant role in increasing the risk of mental health problems in students with PMPU than in those without PMPU.

## 1. Introduction

### 1.1. Problematic Mobile Phone Use

There has also been rapid development and increasingly widespread use of mobile phones. Despite its advantages of convenience and practicability, excessive use has been associated with potential risks in people’s life. Problematic mobile phone use (PMPU) is not a new term and it is defined as an inability to regulate one’s use of the mobile phone, which involves negative consequences in daily life [1]. The prevalence of PMPU does not seem to be negligible, for example, it affects 16% of middle school students in Korean [2] and 26% in a Tunisian population [3].

### 1.2. Problematic Mobile Phone Use and Mental Health

The incidence of mental health problems has increased worldwide [4]. These observations have raised concerns about the adverse effects of excessive mobile phone use on the physical and mental health of college students. Researchers have reported a prospective relationship between mobile phone use and psychological symptoms in college students, and a possible model for this association has been proposed [5], which suggested that depression and sleep disorders were the consequences of high rates of information and communication technology use. It has proved that PMPU was correlated to anxiety or insomnia [6], depression [7], and psychological distress [8] in adolescents or college students. However, there are several other factors that should be considered in epidemiological studies that may influence associations between mobile phone exposure and mental health.

### 1.3. Problematic Mobile Phone Use and Sleep

Sleep disturbances (e.g., delayed sleep phase, sleep duration, sleep patterns, chronotype, sleep quality) among adolescents are closely associated with mobile phone use. Short sleep duration during the week showed higher problematic usage [9]. Those who used their mobile phones more frequently after lights out reported a significantly poorer sleep quality, more fatigue and insomnia symptoms [10]. Mobile phone use for calling and texting after lights out was associated with sleep disturbances (short sleep duration, subjective poor sleep quality, excessive daytime sleepiness, and insomnia symptoms) [11]. Results showed that Composite Scale of Morningness (as a measure of chronotype) scores were the best predictor for problematic mobile phone usage, and as a consequence, evening-oriented university students scored higher on the Mobile Phone Problem Usage Scale [12]. The sleep quality worsened with an increasing level of excessive mobile phone use [13]. As an unstructured leisure activity, mobile phone use with no fixed starting and stopping point may increase the risk of extending and taking up more time, and thus other possible activities and sleep were displaced [14].

### 1.4. The Possible Role of Sleep for Mental Health

Sleep is recognized as necessary for health and overall growth. Sleep deprivation was associated with psychological symptoms, such as negative emotions and depressive symptoms [15]. A large population-based study suggested that higher levels of depression and anxiety were more common in Norway adolescents with a delayed sleep phase [16]. Furthermore, a longitudinal survey suggested there may be a causality between sleep pattern and mental health in adolescents, which indicated that a late bedtime and short sleep duration predicted consequential anxiety and depression [17]. As such, it was effective to develop adolescent health education programs or interventions focusing on sleep quality to improve mental health [17]. A survey based on a national sample showed that US adolescents with insomnia were more at risk for having mental disorders, including mood and anxiety disorders, and poor perceived mental health [18]. Evening chronotype was reported to be associated with depression [19]. The mechanism through which short sleep has an impact on emotional and behavioral functioning in adolescents may involve an increase in negative mood and a decrease in the ability to regulate emotions [14].

### 1.5. Potential Role of Sleep for the Associations between Problematic Mobile Phone Use and Mental Health

Whether there are factors that moderate the associations between PMPU and adolescent mental health has not been well examined. Sleep disturbance was a major risk factor for adolescent mental health, and also influences the association between addictive behaviors and psychological symptoms. At the same time, PMPU was related to sleep problems. A study reported that severe mobile phone use in Finnish girls was related to poor self-reported health, which was directly through poor sleep quality and daytime fatigue [20]. Results from Adams and Kisler supported the mediation hypothesis that sleep disturbance may mediate the relationship between electronic media use and depressive symptoms/anxiety in a sample of college students [21]. Therefore, the interactions of PMPU and sleep quality with mental health need further study. Above all, the study aimed to examine PMPU and its association with mental health in Chinese college students. Furthermore, we investigated how sleep quality influences this association.

## 2. Methods

### 2.1. Participants

A cross-sectional survey was conducted in October 2013 to examine the health and well-being of college students in Anhui, China. Self-completion questionnaires were administered in the classrooms of each participating college major. Cluster sampling was used, with the school department as the primary sampling unit. Between November and December 2013, 4915 questionnaires were distributed. However, we received 4858 completed questionnaires (response rate: 98.8%), as some students were absent. Owing to some responses, a total of 4747 respondents (58.4% female) with a mean age of 19.24 (Standard Deviation (SD) = 1.41) were recruited in the final analysis.

All subjects gave their informed consent for inclusion before they participated in the study. The study was conducted in accordance with the Declaration of Helsinki, and the study was approved by the Ethics Committee of Anhui Medical University (No. 20131196).

### 2.2. Procedure

Data collection was completed between October and December 2013. Teachers and professional investigators distributed questionnaires to the students and instructed them to complete the questionnaires anonymously within 20–30 min in classroom settings. Voluntary cooperation principles were obtained from all participants before the survey.

### 2.3. Instruments

#### 2.3.1. Demographic Factors

Participants provided details about their sex, age, residential background, siblings, family income, tobacco and alcohol use, and internet addiction. We included two questions from the Young Risk Behavior Surveillance System questionnaire [22]. To measure cigarette use, we asked “How many days of the past month did you smoke?” Cigarette users were defined as those smoking on at least one day during the past month. To measure alcohol use, we asked “How many days of the past month did you have at least one drink?” Alcohol users were defined as those drinking on at least one day during the past month. We used Young’s 20-item Internet Addiction Test (YIAT) [23] to assess Internet addiction. Scores of each item range from 1 to 5 (1 = not at all, 2 = occasionally, 3 = frequently, 4 = often, and 5 = always)—total scores range from 20 to 100. A YIAT score greater than 50 was defined as internet addiction [24]. Cronbach’s alpha and split-half reliability coefficients were 0.90 and 0.86, respectively.

#### 2.3.2. Problematic Mobile Phone Use

To evaluate PMPU, we used the Self-rating Questionnaire for Adolescent Problematic Mobile Phone Use (SQAPMPU), which was a standardized instrument and suitable for use with college students [25]. It was comprised of 13 items, with three dimensions named withdrawal symptoms, craving, and physical and mental health status. Example items included, “My leisure activities are reduced due to the time I spend on my mobile phone,” “I become irritable if I have to switch off my mobile phone for meetings, dinner engagements, or at the movies.” and “I need to spend more time on my mobile phone to be satisfied.” Each item scored from 1 (Not true at all) to 5 (Extremely true), based on a five-point Likert scale [26]. The variance cumulative contribution rate was 59.13%. Cronbach’s alpha coefficient was 0.87. Total scores ranged from 13 to 65, using the 75th percentile as the cutoff point. As such, PMPU behaviors were categorized as “No” (<*P*_75_) or “Yes” (≥*P*_75_).

#### 2.3.3. Sleep Quality

The Pittsburgh Sleep Quality Index (PSQI) [27] is a self-rated scale that assesses sleep quality during the past month. The scale contains 19 items, which covered seven components named subjective sleep quality, sleep latency, sleep duration, habitual sleep efficiency, sleep disturbances, use of sleep medication, and daytime dysfunction. Subjective sleep quality referred to perceived overall sleep quality. Sleep latency measures how long it took to fall asleep. Sleep duration referred to the actual length of sleep. Habitual sleep efficiency was calculated by the number of hours slept and the number of hours spent in bed. Sleep disturbances referred to some behaviors that negatively affect sleep, such as waking up at late night or early in the morning, getting up at night to use the bathroom, uncomfortable breathing, coughing or snoring loudly, feeling too hot or too cold, having nightmares, and having pain. Each component scored from 0 (no difficulty) to 3 (severe difficulty). Cronbach’s alpha coefficient was 0.729 in the present study. A total score was totaled from the seven component scores, ranging from 0 to 21, with the score of 7 used as the cut-off point [28]. A total score less than or equal to 7 was recognized as good sleep, while a score more than 7 implied poor sleep.

#### 2.3.4. Psychopathological Symptoms

The Multidimensional Sub-health Questionnaire of Adolescents (MSQA) was a multidimensional self-report instrument to measure physical and psychological symptoms [29] which consisted of 71 items. A subset of 39 items was for psychopathological symptoms, which were categorized into three dimensions: emotional symptoms (e.g., anxiety and depressive symptoms), behavioral symptoms (e.g., hostility, out of control, and lack of concentration), and social adaptation problems (e.g., poor interpersonal relationships with schoolmates, families, or friends). Each item had six answer categories, based on the duration of each symptom (<7 days; 7 days–14 days; 15 days–30 days; 31 days–60 days; 61 days–90 days; and >90 days). Cronbach’s alpha coefficient was 0.939 in the present study. In our study, the duration of symptom less than 1 month were recognized as 0, and symptom lasted more than 30 days were recognized as 1. Respondents with a total score of 8 or more were defined as having psychopathological symptoms.

#### 2.3.5. Anxiety Symptoms

To estimate anxiety symptoms, we used the Self-rating Anxiety Scale (SAS) [30], which has been tested for reliability and validity all over the world [31,32,33]. The SAS is comprised of 20 items and the answers were based on a four-point Likert scale: 1 = a little of the time, 2 = some of the time, 3 = good part of the time, 4 = most of the time. Cronbach's alpha coefficient was 0.818 in the present study. A higher score indicated more severe anxiety symptoms. SAS total scores were categorized into two groups using a cut-off score of 50. As such, SAS scores ≥50 reflected the experience of anxiety symptoms [34].

#### 2.3.6. Depressive Symptoms

The Center for Epidemiologic Studies Depression Scale (CES-D) [35] was used to assess depressive symptoms during the past week. It is comprised of 20 questions and has four answer categories with scores from 0 to 3: rarely or none of the time/<1 day (0), some or a little of the time/1–2 days (1), occasionally or a moderate amount of the time/3–4 days (2), and most or all of the time/5–7 days (3). Cronbach’s alpha coefficient was 0.87 in the present study. CES-D scores ≥20 in accordance with the Chinese norm was defined as the experience of depression symptoms [36].

### 2.4. Statistical Analysis

All statistical analyses were conducted using SPSS version 10.0 (SPSS Inc., Chicago, IL, USA). Frequencies and percentages for categorical variables, and mean and SD for continuous variables were used in descriptive analysis. Chi-square tests were conducted to examine the prevalence of mental health problems among students grouped according to PMPU and sleep quality. Multivariate logistic regression analyses were employed to examine the independent and interactive effects of PMPU and sleep quality with mental health, adjusting for confounding factors. According to Table 1, not all confounding factors were significant for mental health, thus we did not include all confounding factors in Table 2. We have detected the multi-collinearity for the variables, and the results showed that the variance inflation factors (VIF) were less than 2, the values of tolerance were less than 1, and the condition index was less than 4, which indicated that multicollinearity was not existent. *p*-values < 0.05 (two-tailed) were considered as statistically significant.

## 3. Results

### 3.1. Characteristics of the Sample

Sample characteristics were displayed in Table 1. There were responses from 4747 students (41.6% male, *n* = 1973). We observed PMPU in 28.2% and poor sleep quality in 9.8% of participants. Psychopathological symptoms were more common in male individuals (16.2% of female individuals vs. 18.9% of male individuals, *p* < 0.05). However, there was a near sex-based significance for anxiety (*p* = 0.065) and the results were not being significantly different for depressive symptoms (*p* = 0.594). Students reporting low family income showed higher rates of poor mental health. Furthermore, students who were current smokers reported higher rates of psychopathological symptoms than non-smokers (23.4% vs. 17.0%, respectively, *p* < 0.01) and higher rates of anxiety symptoms (25.5% vs. 15.7%, respectively, *p* < 0.001). Anxiety symptoms also appeared to be higher among students who were current drinkers than those who were not (18.9% vs. 14.8%, respectively, *p* < 0.001). Higher rates of psychopathological, depression and anxiety symptoms were also seen in those with Internet addiction (IA), PMPU, and poor sleep quality (all *p* < 0.001).

### 3.2. Associations of PMPU and Sleep Quality with Mental Health 

There was a positive association of PMPU and sleep quality with mental health symptoms (Table 2). Logistic regression showed that both PMPU and sleep quality are independently associated with mental health symptoms. Adjusted models suggested that PMPU was related to psychopathological symptoms (odds ratio [OR]: 2.46, 95% confidence interval [CI]: 2.06–2.94), anxiety symptoms (OR: 2.02, 95% CI: 1.68–2.43), and depressive symptoms (OR: 2.53, 95% CI: 2.10–3.05). Additionally, poor sleep quality was positively correlated with psychopathological symptoms (OR: 3.34, 95% CI: 2.67–4.16), anxiety symptoms (OR: 3.72, 95% CI: 2.99–4.62), and depressive symptoms (OR: 4.97, 95% CI: 3.99–6.19).

### 3.3. Interactions of PMPU and Sleep Quality with Mental Health 

The results of a regression analysis examining the interactions of PMPU and sleep quality with mental health were shown in Table 3. There was a significant interaction of PMPU and sleep quality with mental health symptoms (*p* < 0.001). Table 3 presents crude and adjusted OR (95% CI) for psychopathological symptoms, anxiety symptoms, and depressive symptoms in those with PMPU or poor quality sleep compared with the reference group (no PMPU or poor sleep quality). OR (95% CI) for psychopathological symptoms, anxiety symptoms, and depressive symptoms were 7.60 (5.55–10.41), 6.68 (4.89–9.13), and 11.28 (8.21–15.50), respectively. PMPU students with poor sleep quality were more likely to be with mental health symptoms.

## 4. Discussion

This study was one of few to investigate PMPU in Chinese college students. The results provided evidence of the association between PMPU and poor mental health among college students. The results suggested that PMPU was positively related to psychopathological symptoms, anxiety, and depression. In addition, our results indicated that sleep quality was also positively correlated with mental health. Furthermore, we confirmed interactions of PMPU and poor sleep quality with mental health among Chinese college students.

We observed PMPU in 28.2% of participants, which was higher than in previous studies [37]. This may be because our sample was college students, whereas the other study investigated high school students. Furthermore, the evaluation criterion for PMPU differed. Sánchez-Martínez and Otero [37] used two forced choice (yes/no) questions, reporting an estimated prevalence of mobile phone dependence of 20%. They used direct judgment methods [37], whereas we considered total scores and percentiles. We might therefore expect our results to differ.

To our knowledge, several studies supported the existence of the relationship between PMPU and psychological symptoms. An explorative prospective study reported women with high combined use of computers and mobile phones at baseline showed an increased risk of depression in a one-year follow-up. Furthermore, text message use was also related to depressive symptoms in men [5]. Augner and Hacker [38] reported that scores of depression were positively correlated with PMPU scores. The association between depression symptoms and intensive cell phone use has been confirmed elsewhere [37]. Mobile phone use has also been reported as a diversion to kill time or avoid some other activity by anxious individuals [39], whilst it has been proven that excessive mobile phone use was connected with a high risk of anxiety and insomnia [6]. General psychological distress has been shown to be related to abnormal use of both the internet and mobile phones [8]. Another study indicated that those with excessive mobile phone use not only experienced higher levels of depression and interpersonal anxiety [40]. In addition, depressed adolescents are at higher risk of PMPU after controlling for the confounding effects of sex, age, and residential area [7].

Consistent with our hypothesis, we found that PMPU was related to sleep quality. Using the mobile phone after going to bed led to increasing sleep problems [41]. The total score of sleep quality showed a direct significant association with cell-phone overuse score [42]. Daytime dysfunction scores were higher in the high smartphone use and positive correlations were found between the Smartphone Addiction Scale scores and PSQI scores in university students [43]. However, few studies have examined the association between PMPU and sleep. A hypothesis was that many technologies such as computers, televisions, and phones could emit shortwave light. When many adolescents use mobile phones at night, artificial shortwave light exposure could affect sleep and neurobehavioral functions, and chronic inopportune exposure to shortwave light causes a malfunction of the circadian timing system, which results in sleep problems and depressive symptoms [44]. The mechanisms underlying this association remain to be explained. Future studies should reveal more information about these mechanisms.

Poor sleep quality can cause mental problems. Adolescents may suffer depressive symptoms exacerbated by poor sleep. A large population-based study also reported a significant interaction of insomnia and sleep duration with depression, which indicated a greater than eightfold increase in odds ratios of depression in Norwegian adolescents aged 16–18 years with insomnia who slept <6 h [45]. Similarly, a Chinese cross-sectional survey showed that sleep disturbance was more prevalent among Chinese adolescents with depressive symptoms [46]. Additionally, reduced sleep has been found to be associated with increased anxiety scores [47]. There were changes in sleep patterns during the transition to college, and the authors considered potential cross-lagged associations between adolescents’ sleep and anxiety and depressive symptoms. A prevention of depression is by improving adolescents’ sleep quality, as there is evidence that disturbed sleep is a factor for the development of depressive symptoms during adolescence [48].

We have found that poor sleep quality was an independent risk of mental health. Furthermore, the association between PMPU and mental health was less significant in adolescents who had a higher sleep quality. The results raised the possibility that good sleep quality may reduce the risk of mental health among adolescents with PMPU. Studies that obviously examined the mediation of sleep disturbance for the relationship between PMPU and mental health were rare. Our findings supported that the relationships between PMPU and mental health were mediated by sleep disturbance, which were in line with Lemola et al., who implied that sleep disturbance partially mediated the relationship between electronic media use in bed before sleep and symptoms of depression [14]. There was evidence that maintaining healthy sleep patterns may possibly reduce the incidence of mental disorders in college students with PMPU. Understanding these relationships may provide a basis for developing strategies to prevent mental health problems in adolescents with PMPU, and thus warrants further study.

### Limitations

Several limitations should be considered when interpreting our results. First, as this was a cross-sectional study, causality cannot be confirmed. Second, students answered questionnaires anonymously, which may increase the students’ arbitrary disclosure of symptoms. Third, self-reporting questionnaires may lead to recall bias. Finally, the findings may not be generalized to all Chinese students since only medical college students were included. Future research, particularly cohort studies, are essential for determining the direction of causality between PMPU and mental health problems, and to better understand the influence of sleep quality on this relationship. Nonetheless, whether mental health is a cause or effect of PMPU is worthy of more discussion.

## 5. Conclusions

We found cross-sectional associations between PMPU and mental health problems, with interactions of PMPU and sleep quality on mental health in adolescents. The study highlights that poor sleep quality may play a more significant role in increasing the risk of mental health problems in students with PMPU than in those without PMPU. Based on these results, we suggest that improving sleep quality may be an effective strategy to decreasing the risk of mental health problems among adolescents with PMPU.

## Figures and Tables

**Table 1:** Sample characteristics.

Variables	Total	Psychopathological Symptoms		Anxiety Symptoms		Depressive Symptoms	
		*n* (%)/M ± SD	*p*-Value	*n* (%)/M ± SD	*p*-Value	*n* (%)/M ± SD	*p*-Value
**Age**	19.24 ± 1.41	19.20 ± 1.42		19.59 ± 1.44		19.34 ± 1.46	
**Sex**							
Male	1973 (41.6)	373 (18.9)	0.016	344 (17.4)	0.065	320 (16.2)	0.594
Female	2774 (58.4)	450 (16.2)		428 (15.4)		434 (15.6)	
**Residential area**							
Rural	2680 (56.5)	447 (16.7)	0.173	458 (17.1)	0.079	429 (16.0)	0.791
Urban	2067 (43.5)	376 (18.2)		314 (15.2)		325 (15.7)	
**Any siblings**							
Yes	3133 (66.0)	539 (17.2)	0.735	524 (16.7)	0.229	500 (16.0)	0.843
No	1614 (34.0)	284 (17.6)		248 (15.4)		254 (15.7)	
**Perceived family income**							
Low	1490 (31.4)	310 (20.8)	<0.001	295 (19.8)	<0.001	295 (19.8)	<0.001
Medium	3032 (63.9)	473 (15.6)		445 (14.7)		428 (14.1)	
High	225 (4.7)	40 (17.8)		32 (14.2)		31 (13.8)	
**Cigarette use**							
Yes	282 (5.9)	66 (23.4)	0.006	72 (25.5)	<0.001	50 (17.7)	0.382
No	4465 (94.1)	757 (17.0)		700 (15.7)		704 (15.8)	
**Alcohol use**							
Yes	1717 (36.2)	319 (18.6)	0.089	324 (18.9)	<0.001	281 (16.4)	0.494
No	3030 (63.8)	504 (16.6)		448 (14.8)		473 (15.6)	
**Internet addiction**							
Yes	543 (11.4)	270 (49.7)	<0.001	209 (38.5)	<0.001	242 (44.6)	<0.001
No	4204 (88.6)	553 (13.2)		563 (13.4)		512 (12.2)	
**PMPU**							
Yes	1340 (28.2)	447 (33.4)	<0.001	378 (28.2)	<0.001	415 (31.0)	<0.001
No	3407 (71.8)	376 (11.0)		394 (11.6)		339 (10.0)	
**Sleep quality**							
Poor	464 (9.8)	197 (42.5)	<0.001	196 (42.2)	<0.001	218 (47.0)	<0.001
Good	4283 (90.2)	626 (14.6)		576 (13.4)		536 (12.5)	

*n*: Sample size; M: Mean; SD: Standard Deviation.

**Table 2:** Relationship of problematic mobile phone use (PMPU) and sleep quality with mental health.

Variables	Psychopathological Symptoms		Anxiety Symptoms		Depressive Symptoms	
	*Crude OR* (95% CI)	*Adjusted OR* (95% CI) ^a^	*Crude OR* (95% CI)	*Adjusted OR* (95% CI) ^b^	*Crude OR* (95% CI)	*Adjusted OR* (95% CI) ^c^
**PMPU**						
Yes	3.69 (3.15–4.33) **	2.46 (2.06–2.94) **	2.69 (2.28–3.16) **	2.02 (1.68–2.43) **	3.66 (3.10–4.33) **	2.53 (2.10–3.05) **
No	1.00	1.00	1.00	1.00	1.00	1.00
**Sleep Quality**						
Poor	3.61 (2.92–4.47) **	3.34 (2.67–4.16) **	4.06 (3.29–5.01) **	3.72 (2.99–4.62) **	5.35 (4.32–6.62) **	4.97 (3.99–6.19) **
Good	1.00	1.00	1.00	1.00	1.00	1.00

* *p* < 0.05; ** *p* < 0.001. ^a^ adjusted for sex, perceived family income, cigarette use, and Internet addiction. ^b^ adjusted for age, perceived family income, cigarette use, alcohol use, and Internet addiction. ^c^ adjusted for age, perceived family income, and Internet addiction.

**Table 3:** Interactions of PMPU and sleep quality with mental health.

PMPU	Sleep Quality	Psychopathological Symptoms		Anxiety Symptoms		Depressive Symptoms	
		*Crude OR* (95% CI)	*Adjusted OR* (95% CI) ^a^	*Crude OR* (95% CI)	*Adjusted OR* (95% CI) ^b^	*Crude OR* (95% CI)	*Adjusted OR* (95% CI) ^c^
Yes	Poor	12.38 (9.23–16.61) **	7.60 (5.55–10.41) ^a^^,^**	9.81 (7.33–13.12) **	6.68 (4.89–9.13) ^b,^**	17.58 (13.01–23.75) **	11.28 (8.21–15.50) ^c,^**
	Good	3.84 (3.22–4.57) **	2.56 (2.10–3.10) ^a,^**	2.85 (2.38–3.42) **	2.16 (1.77–2.65) ^b,^**	3.91 (3.24–4.71) **	2.70 (2.20–3.31) ^c,^**
No	Poor	4.05 (3.01–5.45) **	3.71 (2.74–5.03) ^a,^**	4.74 (3.55–6.32) **	4.40 (3.27–5.92) ^b,^**	6.28 (4.69–8.40) **	5.79 (4.31–7.78) ^c,^**
	Good	1.00	1.00	1.00	1.00	1.00	1.00

* *p* < 0.05; ** *p* < 0.001. ^a^ adjusted for sex, perceived family income, cigarette use, and Internet addiction. ^b^ adjusted for age, perceived family income, cigarette use, alcohol use, and Internet addiction. ^c^ adjusted for age, perceived family income, and Internet addiction.
